# Resection Margins for Colorectal Metastases to The Liver: Do They Make A Difference?

**DOI:** 10.1155/1996/95180

**Published:** 1996

**Authors:** Reid B. Adams, Bernard Langer

**Affiliations:** 1 Department of Surgery Univeristy of Virginia Health Sciences Center Charlottesville Virginia USA; 2 University of Toronto & The Toronto Hospital Toronto Canada

## Abstract

Objective: The authors determined an appropriate surgical treatment for liver metastases from colorectal cancers. Clinicopathologic featuresof metastatic lesions of colorectal cancers were studied.

Summary Background Data: Major hepatic resection is the usual procedure for treatment of hepatic metastases from colorectal cancers.

Methods: Forty consecutive patients who underwent hepatic resections were prospectively studied, for a total of 89 metastatic liver tumors.

Results: Metastatic tumor often extended along Glisson’s capsule, including invasion to the portal vein (9 cases), the hepatic vein (3 cases), the bile duct (16 cases), and the nerve (6 cases). The main tumor had small satellite nodules in only one patient, and there were no microscopic deposits in the parenchyma, even within 10 mm from the metastatic tumors. Fibrous pseudocapsule formation was observed in 28 patients.

Discussion: The rarity of intrahepatic metastasis from metastatic tumor supports nonanatomic limited hepatic resection as the procedure of choice for metastatic colorectal cancer in the liver. The spread via Glisson's capsule should be taken into consideration for complete tumor clearance.


bile ducts appear to bear the brunt of the reperfusion injury.

The experiments described by Noack *et al.* can obviously be extended to other transplantable solid organs. For example, the relative susceptibility of glomerular cells and renal tubular cells to either anoxia or reoxygenation would be of interest. Other organs affected by disease processes in which the reperfusion injury has been implicated can also be studied using the techniques described above.

### REFERENCES

1. Jamieson, N.V., Lindell, S., Sundberg, R., Southard, J.H. and Belzer, F.O. (1988) An analysis of the components of UW solution using the isolated perfused rabbit liver. *Transplantation*, **46**, 512–516.
2. Kalayoglu, M., Sollinger, H.W. and Stratta, R.J., *et al.* (1988) Extended preservation of the liver for clinical transplantation. *Lancet*, **1**, 617–619.
3. Thurman, R.G., Marzi I., Seitz , G., Thies, J., Lemasters, J.J. and Zimmerman, F.A. (1988) Hepatic reperfusion injury following orthotopic liver transplantation in the rat. *Transplantation*, **46**, 502.
4. Petrowsky, H., Dippe, B. and Beck. P., *et al.* (1995) Do oxygen radicals play a role in primary dysfunction transplanted livers following preservation in University of Wisconsin solution? *Transplant. Proc.*, **27**, 729–731.
5. Sanchez-Urdazpal, L., Gores, G.J. and Lemasters, J.J., *et al.* (1993) Carolina Rinse solution decreases liver injury during clinical liver transplantation. *Transplant. Proc.*, **25**, 1574–1575.
6. Ishii, M., Vroman, B. and LaRusso, N.F. (1989) Isolation and morphologic characterization of bile duct epithelial cells from normal rat liver. *Gastroenterology* **97**, 1236.
7. Caraceni, P., Gasbarrini, A. and Rya, H.S., *et al.* (1994) Human hepatocytes differ from rat hepatocytes in their sensitivity to anoxia-reperfusion injury. *Transplant. Proc.*, **26**, 3307–3308.
8. Emre, S., Schwartz, M.E. and Mor, E., *et al.* (1994) Obviation of prereperfusion rinsing and decrease in preservation/reperfusion injury in liver transplantation by portal blood flushing. *Transplanation*, **57**, 799–803.
9. Sanchez-Urdazpal, L., Gores, G.J. and Ward, E.M., *et al.* (1992) Ischaemic-type biliary complications after orthotopic liver transplantation. *Hepatology*, **16**, 49–53.
10. Menegaux, F., Egawa, H., Keeffe, E.B., So, S.K., Concepcion, W., Collins, G.M. and Esquivel, C.O. (1993) Comparative effects of blood, Colloid, and Ringer's lactate terminal allograft rinse on the results of orthotopic liver transplantation. *Transplant. Proc.*, **25**, 3196–3198.

Professor D. Kahn
Head: Organ Transplant Unit
Department of Surgery
University of Cape Town Medical School
Observatory 7925
Cape Town
South Africa

### RESECTION MARGINS FOR COLORECTAL METASTASES TO THE LIVER: DO THEY MAKE A DIFFERENCE?

### ABSTRACT

*Yamamoto, J., Sugihara, K., Kosuge, T., Takayama, T., Shimada, K., Yamasaki, S., Sakamoto, M. and Hirohashi, S. (1995) Pathologic support for limited hepatectomy in the treatment of liver metastases from colorectal cancer. Annals of Surgery, 221*: 74–78

### Objective

**The authors determined an appropriate surgical treatment for liver metastases from colorectal cancers. Clinicopathologic features of metastatic lesions of colorectal cancers were studied.**

### Summary Background Data

**Major hepatic resection is the usual procedure for treatment of hepatic metastases from colorectal cancers.**

**Methods**

**Forty consecutive patients who underwent hepatic resections were prospectively studied, for a total of 89 metastatic liver tumors.**

**Results**

**Metastatic tumor often extended along Glisson's capsule, including invasion to the portal vein (9 cases), the hepatic vein (3 cases), the bile duct (16 cases), and the nerve (6 cases). The main tumor had small satellite nodules in only one patient, and there were no microscopic deposits in the parenchyma, even within 10 mm from the metastatic tumors. Fibrous pseudocapsule formation was observed in 28 patients.**

**Discussion**

**The rarity of intrahepatic metastasis from metastatic tumor supports nonanatomic limited hepatic resection as the procedure of choice for metastatic colorectal cancer in the liver. The spread via Glisson's capsule should be taken into consideration for complete tumor clearance.**

KEY WORDS: Hepatic resection secondary colorectal cancer liver metastases.

### PAPER DISCUSSION

Evolution of therapy for colorectal metastases confined solely to the liver has resulted in surgical resection as the best available therapeutic approach for these lesions. This has occurred because alternative therapies have little to no effect on the natural history of hepatic metastases, while surgical resection offers a good chance for cure while becoming increasingly safe. Reported series of surgical resection for hepatic metastases from colorectal carcinoma with intent to cure demonstrate a 5 year survival rate of 30–40%[1–3]. Recently significant 10 to 20 year survival following hepatic resection has been reported[2]. Improved understanding of hepatic segmental and vascular anatomy along with meticulous technique has contributed to a decrease in operative mortality rates to less than 2% in referral centers. This paper by Yamamoto *et al.* seeks to refine surgical therapy through a careful study of pathologic findings in resected hepatic colorectal metastasis.

Forty patients over a 1 year period (1991–1992) underwent conservative liver resection of a total of 89 colorectal metastases which were examined macroscopically and microscopically. Equal numbers of patients had solitary and multiple metastases. Nine patients (23%) had gross invasion into "Glisson's capsule" (portal pedicles, also known as vasculo-biliary sheaths of Walaeus)[4]; 8 had bile duct invasion, 1 had neural invasion. Microscopic invasion of portal pedicles was seen in 22 of 40 (55%) cases. Bile duct invasion accounted for 40%, portal vein 23%, nerve 15%, and hepatic vein 8% of these cases. Proximal tumour extension along the portal triads was common, occurring in 9 of 40 (23%) of cases. Tumor extended up to 23 mm from the edge of the main tumor, the average extension in these 9 cases occurring 12 mm from the tumor edge. A fibrous pseudocapsule surrounding the tumor was absent in 30% of cases, thin ( several layers of collagen bundles present) in 33%, and thick (ten or more collagen bundles present) in 38%. A satellite lesion close to the main metastasis was seen in only one patient. Positive surgical margins were identified in 20% of patients.

The authors present these findings as supportive of their approach of "non anatomical limited resection" without the need for the usually accepted minimum of one centimeter tumor free margin. They go on to suggest that one "can remove a tumor by shaving the non cancerous liver tissue, especially when it has a thick fibrous pseudocapsule." This approach seems inappropriate since their own data shows that this approach has resulted in 20% of their patients having positive surgical margins. The data in the literature in much larger series of cases lends strong support to the opposite view, that positive margins are uniformly associated with poor outcome and that the best intraoperative predictor for a negative margin is at least one centimeter tumor clearance[1,5–7]. In those reports[1,2,5,6,8] that stratify the width of surgical margin, increasing survival correlates well with increasing margin of resection. Thus, a resection margin of one centimeter is the current recommendation and should remain so until further data dictates otherwise. To make the claim that more conservative resection is safe, the authors would have to provide us with follow up information demonstrating that these narrow (or absent) margins are compatible with long term survival. They have not done so.

This paper does contain some additional information about the route of local spread of metastatic colon cancer which might influence the planning of surgical resection. The authors have found microscopic invasion of adjacent portal pedicles in over half of their patients and proximal extension of tumor in one quarter of cases. Given the extent of such proximal invasion, one would be even less likely to minimize the resection margins than previously, especially more proximally along the portal pedicles. How might this influence the nature of the operation? We would suggest that going more proximally along portal pedicles might mean doing more, no+ fewer anatomical rather than non anatomical resections. The authors, curiously have come to the opposite conclusion.

Finally, what is gained by deliberately taking out less, rather than more liver? If it meant that the operation became safer without compromising the chance for cure, it might be reasonable. Patients with colorectal metastasis usually have normal livers and can tolerate major hepatic resections very well without fear of liver failure. Our own experience also has been that anatomical liver resection is often an easier procedure and associated with less blood loss than complex non anatomical resection. We, therefore, often perform the former in cases where the latter might also give us adequate (≥ 1 cm) tumor free margins.

The careful analytical approach to study of resected specimens that these authors have taken could be a useful one, but this work needs to be extended to larger numbers of patients and reported with long term followup, so that possible relationships may be drawn between pathological features and outcomes. Even that information by itself would not be sufficient to warrant a change in practice until prospective stu-dies of surgical technique demonstrated a measurable significant benefit, either in safety of operation, or in improved survival.

For the present, we must caution readers to adhere to current surgical principles which call for adequate margins (at least 1 cm) in all directions when resecting metastatic colon cancer in the liver. Given the authors findings regarding portal pedicle invasion, particular attention should be paid to getting at least that margin proximally. Non anatomical resection remains an acceptable method of liver resection as long as the above principles are followed and it can be done as safely as anatomical resection.

### REFERENCES

1. Registry of Hepatic Metastases. (1988) Resection of the liver for colorectal carcinoma metastases: A multi-institutional study of indications for resection. *Surgery,* **103**, 278–288.
2. Scheele, J., Stangl, R., Altendorf-Hofmann, A. and Gall, F.P. (1991) Indicators of prognosis after hepatic resection for colorectal secondaries. *Surgery,* **110**, 13–29.
3. Doci, R., Gennari, L., Bignami, F., Montalto, F., Morabito, A. and Bozzetti, F. (1991) One hundred patients with hepatic metastases from colorectal cancer treated by resection: analysis of prognostic determinants. *British Journal of Surgery,* **78**, 797–801.
4. Couinaud, C. (1989) Surgical Anatomy of the Liver Revisited. *Couinaud, C.* Paris.
5. Ekberg, H., Tranberg, K.-G., Andersson, T., Lundstedt, C., Hagerstrand, K., Ranstam, J. and Bengmark, S. (1986) Determinants of survival in liver resection for colorectal secondaries. *British Journal of Surgery,* **73**, 727–731.
6. Cady, B., Stone, M.D., McDermott, W.V., Jenkins, R.L., Bothe, A., Lavin, P.T., Lovett, E.J. and Steele, G.D. (1992) Technical and biological factors in disease-free survival after hepatic resection for colorectal cancer metastases. *Archives of Surgery,* **127**, 561–568.
7. Holm, A., Bradley, E. and Aldrete, J.S. (1989) Hepatic resection of metastases from colorectal carcinoma. Morbidity, mortality and pattern of recurrence. *Annals of Surgery,* **209**, 428–434.
8. Hughes, K., Scheele, J. and Sugarbaker, P.H. (1989) Surgery for colorectal cancer metastatic to the liver. *Surgical Clinics of North American,* **69**, 339–359.

Reid B Adams, MD
Assistant Professor
Department of Surgery
Univeristy of Virginia Health Sciences Center
Charlottesville
Virginia

Bernard Langer, MD
Professor of Surgery
University of Toronto & The Toronto Hospital
Toronto
Canada

